# Effects of exogenous ascorbic acid on seed germination and seedling salt-tolerance of alfalfa

**DOI:** 10.1371/journal.pone.0250926

**Published:** 2021-04-29

**Authors:** Zhao Chen, Xin-long Cao, Jun-peng Niu

**Affiliations:** College of Grassland Agriculture, Northwest A&F University, Yangling China; University of Agriculture, PAKISTAN

## Abstract

Alfalfa (*Medicago sativa* L.) is an important legume crop for forage, agriculture, and environment in the world. Ascorbic acid (AsA) plays positive roles in plants. However, its effects on germination and salt-tolerance of alfalfa are unknown. The effects of AsA applications on seed germination and seedling salt-tolerance of alfalfa were investigated. The results revealed that 0.1 and 1 mmol L^-1^ of exogenous AsA increased germination, amylase, and protease, as well as seedling length, fresh weight (FW), dry weight (DW), and endogenous AsA both in the shoots and roots, except that 1 mmol L^-1^ AsA reduced the activities of α-amylase, β-amylase and protease on day 3. However, 10 and 100 mmol L^-1^ AsA inhibited these parameters and even caused serious rot. It indicates that 0.1 mmol L^-1^ AsA has the optimal effects, whereas 100 mmol L^-1^ AsA has the worst impacts. Another part of the results showed that 0.1 mmol L^-1^ AsA not only enhanced stem elongation, FW and DW, but also increased chlorophyll and carotenoids both under non-stress and 150 mmol L^-1^ NaCl stress. Furthermore, 0.1 mmol L^-1^ AsA mitigated the damages of membrane permeability, malondialdehyde, and excessive reactive oxygen species (ROS) and ions both in the shoots and roots under 150 mmol L^-1^ NaCl stress. Hence, 0.1 mmol L^-1^ AsA improves growth and induces salt-tolerance by inhibiting excessive ROS, down-regulating the ion toxicity and up-regulating the antioxidant system. The principal component analysis included two main components both in the shoots and roots, and it explained the results well. In summary, the optimum concentration of 0.1 mmol L^-1^ AsA can be implemented to improve the seed germination and seedling growth of alfalfa under salt stress.

## Introduction

Soil salinization has become a worldwide problem and it seriously threatens the social resources, population, environment, and food in the world [1–3]. Soil salinization has affected agriculture because salinity is an abiotic factor influencing growth, development, quality, and productivity of many crops, and alfalfa (*Medicago sativa* L.) is no exception [4–6]. Alfalfa is a perennial deep-rooted legume crop with wide distribution and the largest cultivated acreage. And it not only supplies adequate forage for animals but also improves soil fertility, properties, and quality for sustainable agricultural systems [6–9]. In addition, alfalfa is one of the plants for bio-productivity and the restoring of marginal lands. Because it not only produces beneficial fodder but also enriches soil nitrogen through a symbiotic association with *Rhizobium* spp. Bacteria [10]. Salt stress affects alfalfa growth directly or indirectly, even soil salinization has greatly affected alfalfa production in numerous saline-alkali areas in the world [11,12]. Therefore, planting salt-tolerant and salt-resistant alfalfa varieties is the most economical and effective measure to improve and utilize saline-alkali land.

Ascorbic acid (AsA), also known as ascorbate or vitamin C, is a low molecular weight water-soluble antioxidant both in plants and animals. And AsA is a universal non-enzymatic antioxidant having a substantial potential of not only scavenging reactive oxygen species (ROS), but also modulating many fundamental functions in plants both under stress and non-stress conditions. AsA can be absorbed into plants and increase endogenous AsA content. Exogenous application of AsA plays an important role in many plants exposed to different types of abiotic stresses, including salinity [13], drought [14], heat [15], osmotic [16], and ozone [17]. AsA has been used to ameliorate abiotic stresses, and studies that improve plant growth under salt stress are the most common. Moreover, AsA has the potential to partially alleviate the effects of salinity on seed germination of some grass species under optimal temperature regime [18]. What’s more, AsA has low cost, low price, and few side effects. Therefore, AsA is a potential non-enzymatic antioxidant to improve the salt-tolerance of plants.

The application of exogenous AsA has been investigated widely for the stress tolerance/resistance capabilities of numerous plants. However, there are very few studies have been conducted to reveal the effects of AsA on accelerating germination and altering the defense potential of alfalfa against salinity. Therefore, (1) Zhongmu No.1 (*Medicago sativa* L.) was used to evaluate the effects of AsA on seed germination and seedling salt-tolerance; (2) the physiological and biochemical mechanisms were discussed. This study has a certain significance for improving the growth, quality, and yield of alfalfa in salinity soil.

## Materials and methods

### Effects of AsA on the germination of alfalfa seeds

#### Materials and experiment design

The seeds were sterilized with 5% sodium hypochlorite solution for 10 min and rinsed five times with sterile distilled water. They were then immersed in AsA (0.1, 1, 10, or 100 mmol L^-1^) or distilled water (control), respectively. After 24 h, the seeds were thoroughly washed three to five times using sterile distilled water. Uniform-sized seeds were incubated in a petri dish containing two layers of filter paper. Each treatment included three replications and each replication included 50 seeds. The seeds were kept in an incubator at 25°C with a 16-h daily photoperiod (90 μmol m^−2^ s^−1^) and 8-h dark. The seeds were regularly watered, and the germination and rot indices were recorded every day. Mean germination time (MGT), speed of germination (SG), mean daily germination (MDG), and peak value of germination (PV) were evaluated [19,20]. In addition, the rot rate was observed and calculated every day.

#### α-Amylase, β-amylase and protease

On days 1 and 3 during germination, the seeds were used to assess α-amylase, β-amylase, and protease.

The activities of α-amylase and β-amylase were measured with the kit (Shanghai suoqiao biotechnology co. LTD, China).

The seeds were ground and suspended in 200 μL extraction buffer. After shaking at 30°C for 1 h, the reaction was stopped by 150 μL of 30% acetic acid. The reaction mixture was centrifuged, and the supernatants were measured at 410 nm to calculate protease [21].

#### Length and weight

After 8 d, 10 seedlings were collected for the measurements of length, fresh weight (FW), and dry weight (DW).

#### Endogenous AsA

Endogenous AsA was evaluated after 8 d of germination. The reaction system of samples was measured at 534 nm after 90 min at 30°C [22].

### Effects of AsA on the salt-tolerance of alfalfa seedlings

#### Materials and experiment design

The sterilized seeds were immersed in distilled water for 24 h. After 5 d of germination, the uniform-sized seedlings with two cotyledons were transplanted in 12 pots (10 seedlings per pot) using a 3:1:1 mixture of peat, vermiculite and pearlite, respectively. The pots were divided into four groups (three replications per group): control, NaCl, AsA, and AsA+NaCl treatments. All pots were placed in a growth chamber at 25°C with a 16-h daily photoperiod (90 μmol m^−2^ s^−1^) and 8-h dark. They were regularly watered. After 15 d, the AsA and AsA+NaCl groups were watered with 50 mL of 0.1 mmol L^-1^ AsA. After three times of AsA treatment (one time per 2 d), the NaCl and AsA+NaCl groups were watered with 50 mL of 150 mmol L^-1^ NaCl three times (one time per 2 d). The plants continued to grow for 12 d. Whereafter, the parameters were determined as described in the following methods. And the same physiological and biochemical parameter was determined by the leaves of the same part of the plants.

#### Length and weight

Shoots and roots of five plants per pot were collected for the measurements of length, FW, and DW.

#### Chlorophyll a (Chl a), chlorophyll b (Chl b), total chlorophyll (Chl a+b) and total carotenoids (Cx+c)

Fresh leaves were ground in 95% ethanol by the addition of a little silicon dioxide and calcium carbonate. After filtration and dilution, 3 mL extract was measured at 470, 663, and 645 nm [23].

#### The relative conductivity (RC)

RC represented the relative membrane permeability of alfalfa. The electrical conductivity was measured before and after boiling water bath, respectively; then the RC was calculated.

#### Malondialdehyde (MDA)

The fresh sample was homogenized and centrifugated, 2 mL of supernatant was added to 5 mL of 0.5% 2-thiobarbituric acid. The sample was incubated at 95°C for 30 min. Then, the absorbance was measured [24].

#### Hydrogen peroxide (H_2_O_2_)

Fresh tissues were homogenized and centrifugated. The supernatant was added to 0.5 mL of 0.1 mol L^-1^ potassium phosphate buffer (pH 7.0) and 1 mL of 1 mol L^-1^ KI. After 1 h under dark condition, it was measured at 390 nm [25].

#### Superoxide anion radical (O_2_·^¯^)

Fresh tissues were homogenized and centrifuged. After incubation, 17 mmol L^-1^ sulfanilamides and 7 mmol L^-1^ α-naphthylamine were added to the incubation mixture. After reaction at 25°C for 20 min, the absorbance was measured at 530 nm [26].

#### Antioxidant system

Superoxide dismutase (SOD) activity was measured by the method of Beyer and Fridovich [27]. Catalase (CAT) activity was measured by the method of Aebi [28]. Peroxidase (POD) was calculated according to the method of Yadav et al. [29]. Endogenous AsA was measured according to the above method.

#### Ion levels

The ground samples were extracted with 0.5 mmol L^-1^ HNO_3_ by shaking in vials for 48 h. Then the extracts were diluted for Na^+^, K^+^ (Sherwood 410 Flame Photometer, UK) and Cl^-^ (ICS-1100 Ion Chromatograph, USA). Then the levels were calculated [30].

### Statistical analysis

The mean of three replications was used to represent per parameter. Data were evaluated with Variance Analysis using SPSS 20 software. Differences were considered significant at p<0.05.

## Results

### Effects of AsA on germination indices

**Fig 1:**
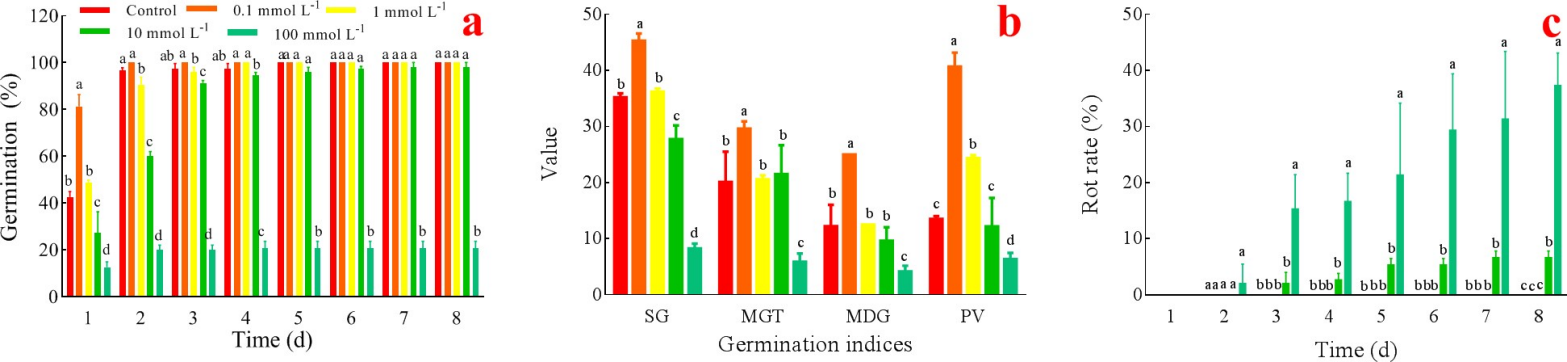
Effects of AsA on germination (a), germination indices (b), and rot rate (c). Lower case letters indicate the difference in each group. The significance level is 0.05, the same below.

It shows that 0.1 mmol L^-1^ AsA significantly increased the germination rate, whereas the higher concentrations of AsA inhibited the germination rate, and 100 mmol L^-1^ AsA had the strongest inhibitory effect (Fig 1A).

It indicates that 0.1 mmol L^-1^ AsA significantly increased SG, MGT, MDG, and PV. Although 1 mmol L^-1^ AsA promoted these parameters, it only occurred a significant difference in PV compared with the control. The higher concentrations of AsA interfered with these parameters; especially 100 mmol L^-1^ AsA had the strongest inhibitory effect (Fig 1B).

The higher concentrations (10 and 100 mmol L^-1^) of AsA caused rot, especially 100 mmol L^-1^ AsA played the worst role. And the seeds treated with 10 or 100 mmol L^-1^ AsA were rotted to 6.67% and 37.33%, respectively (Fig 1C).

### Effects of AsA on α-amylase, β-amylase and protease

**Fig 2:**
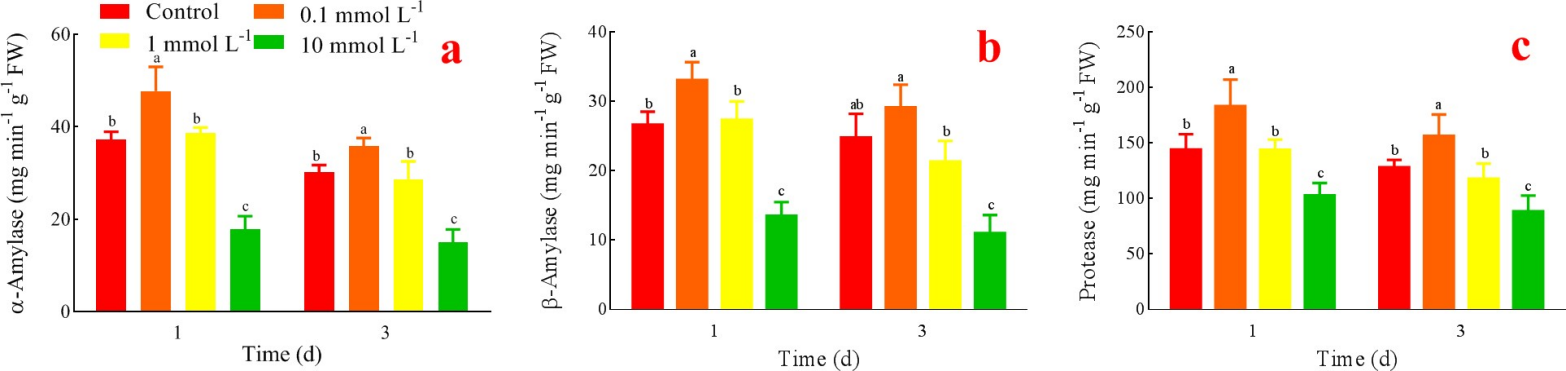
Effects of AsA on α-amylase (a), β-amylase (b), and protease (c).

Compared with the control, α-amylase in the treatment with 0.1 mmol L^-1^ AsA had the highest activity both on days 1 and 3; 1 mmol L^-1^ AsA treatment had no difference, and 10 mmol L^-1^ AsA treatment had the lowest activity (Fig 2A). What’s more, β-amylase (Fig 2B) and protease (Fig 2C) showed similar changes, and 0.1 mmol L^-1^ AsA had positive effects, while the increasing AsA had inhibitory effects.

### Effects of AsA on length, FW and DW of seedlings

**Fig 3:**
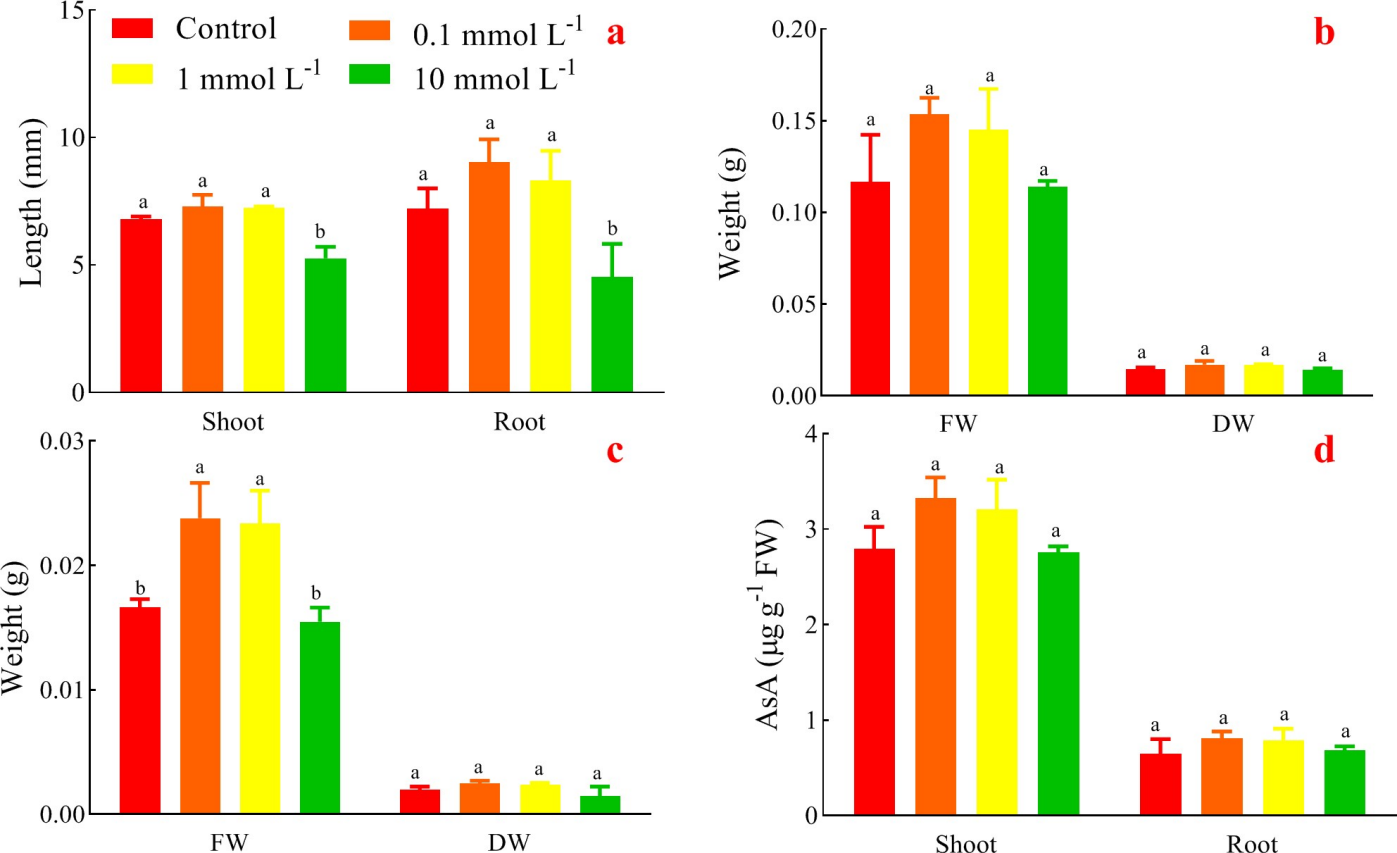
Effects of AsA on shoot length (a), shoot weight (b), root weight (c), and endogenous AsA (d).

The length of shoots and roots of alfalfa treated with 0.1 and 1 mmol L^-1^ AsA were higher than that of the control; whereas 10 mmol L^-1^ AsA remarkably inhibited the elongation of shoots and roots (Fig 3A).

All treatments increased FW and DW of shoots compared with the control, even 0.1 mmol L^-1^ AsA showed the highest values with no significance. What’s more, the promoting effect of 10 mmol L^-1^ AsA was weaker than the other treatments both in FW and DW (Fig 3B).

In addition, 0.1 and 1 mmol L^-1^ AsA significantly increased FW of roots compared with the control, whereas 10 mmol L^-1^ AsA decreased FW of roots (Fig 3C). Meanwhile, 0.1 and 1 mmol L^-1^ AsA increased DW of roots (p >0.05); 10 mmol L^-1^ AsA indistinctively decreased it.

### Effects of AsA on endogenous AsA of seedlings

Exogenous AsA insignificantly increased endogenous AsA both in the shoots and roots (Fig 3D). And 0.1 mmol L^-1^ AsA treatment had the highest endogenous AsA both in the shoots and roots, while the effects of 1 and 10 mmol L^-1^ AsA decreased with the increase of exogenous AsA.

### Effects of AsA on length, FW and DW of the NaCl-treated seedlings

**Fig 4:**
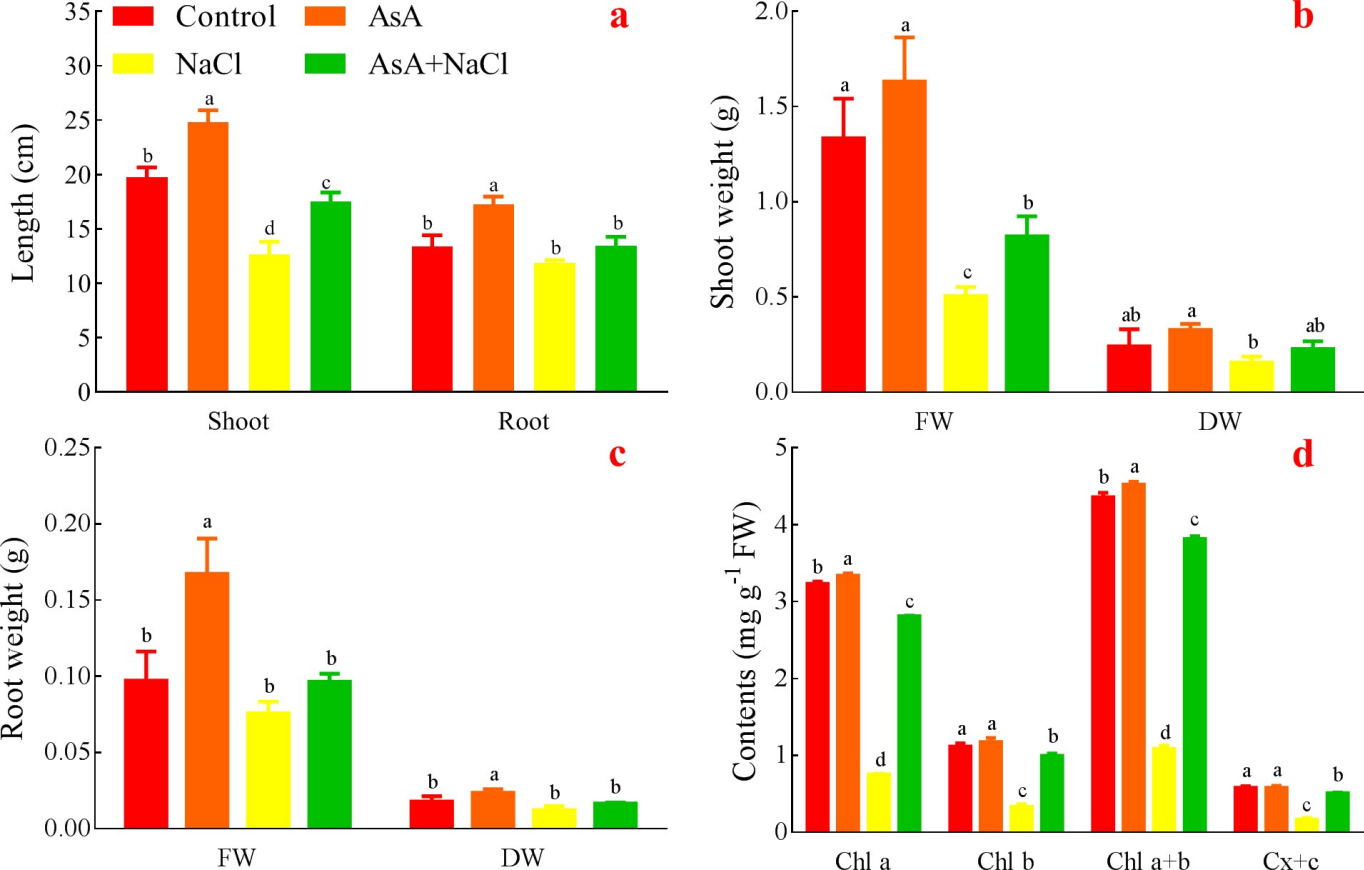
Effects of AsA on shoot length (a), shoot weight (b), root weight (c), and Chl a, Chl b, Chl a+b and Cx+c (d).

The NaCl treatment had the dramatically lowest length whereas the AsA treatment had the dramatically highest length in shoots. The NaCl+AsA treatment remarkably increased shoot length compared with the NaCl treatment. In addition, the roots showed similar results to the shoots, but there was no remarkable difference between the AsA and AsA+NaCl treatments (Fig 4A).

Both in FW and DW of shoots, the AsA treatment had the maximum, whereas the NaCl treatment had the minimum. And the two treatments had significant differences in FW and DW. The weight of the AsA+NaCl treatment was increased, but there was no significant difference compared with that of the NaCl treatment (Fig 4B).

Both FW and DW of roots in the AsA treatment were significantly higher than these of the others. The NaCl treatment had an insignificant minimum; the AsA+NaCl treatment insignificantly increased the weight compared with the NaCl treatment (Fig 4C).

### Effects of AsA on chlorophyll content of the NaCl-treated seedlings

The AsA treatment had the remarkable maximum in Chl a, whereas the NaCl treatment had the remarkable minimum. The AsA+NaCl treatment distinctly increased Chl a compared with the NaCl treatment. Chl b in the NaCl treatment was the distinct lowest and was significantly lower than in the AsA+NaCl treatment. The changes of Chl a+b and Cx+c were similar to Chl a and Chl b, respectively. It indicates that AsA can increase Chl a, Chl b, Chl a+b, and Cx+c under NaCl condition (Fig 4D).

### Effects of AsA on RC, MDA, H_2_O_2_ and O_2_·^¯^of the NaCl-treated seedlings

**Fig 5:**
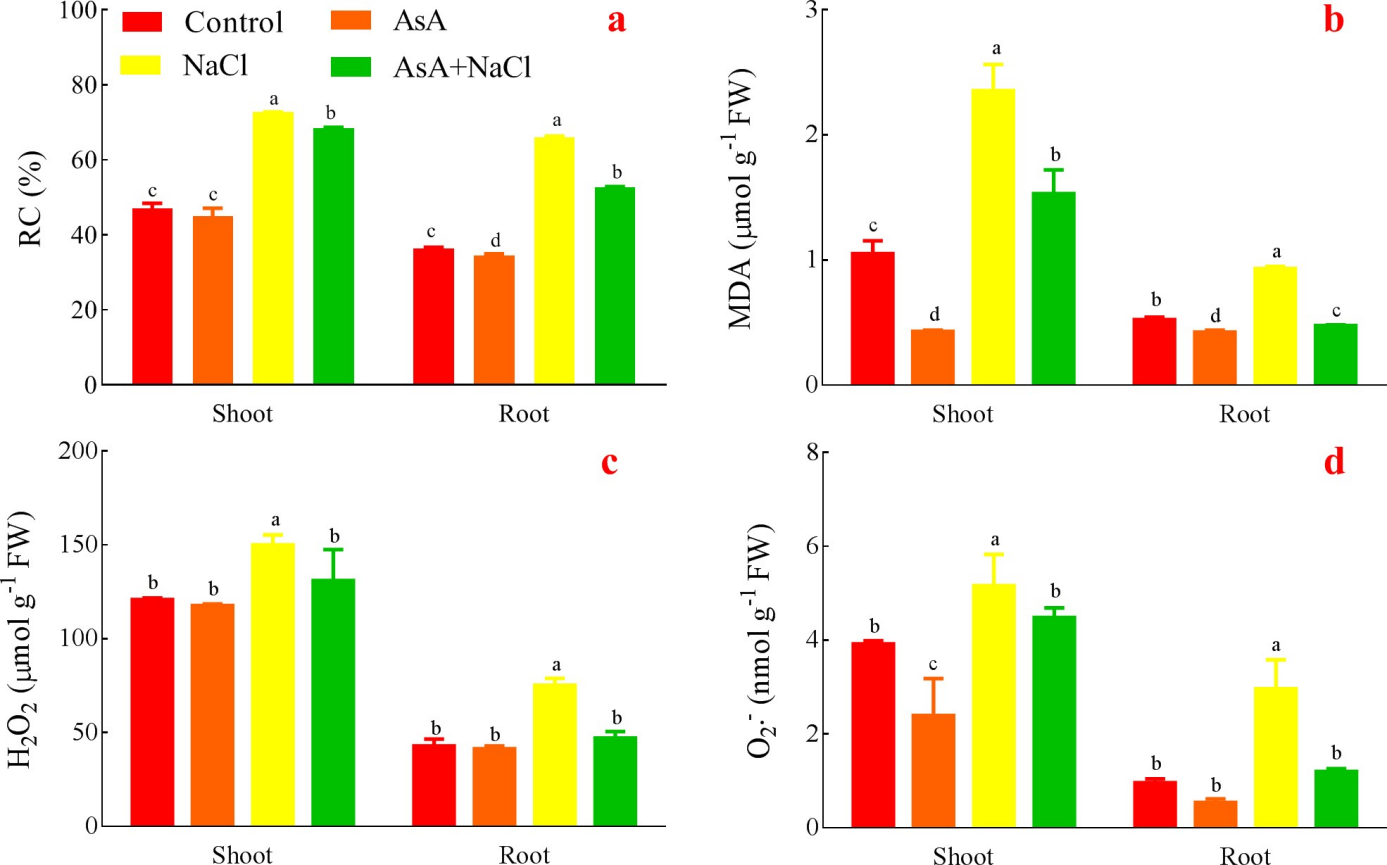
Effects of AsA on RC (a), MDA (b), H_2_O_2_ (c), and O_2_·^¯^ (d).

RC both in the shoots and roots of the NaCl treatment was significantly higher than the others. The AsA+NaCl treatment was lower than the NaCl treatment but dramatically higher than the control and the AsA treatment. What’s more, the AsA treatment significantly had the lowest RC both in the shoots and roots (Fig 5A).

For MDA, the AsA treatment showed a significant minimum both in the shoots and roots, while the NaCl treatment showed a significant maximum. The AsA+NaCl treatment distinctly decreased MDA compared with the NaCl treatment (Fig 5B).

H_2_O_2_ in the AsA treatment was insignificantly inhibited both in the shoots and roots compared with the control. H_2_O_2_ in the NaCl treatment had a remarkable maximum, while the AsA+NaCl treatment remarkably reduced H_2_O_2_ content compared with the NaCl treatment (Fig 5C).

The AsA treatments decreased O_2_·^¯^compared with the control in the shoots and roots. O_2_·^¯^ in the shoots or roots of the NaCl treatment was significantly highest; the AsA treatment had the minimum. In addition, the AsA+NaCl treatment remarkably reduced O_2_·^¯^compared with the NaCl treatment (Fig 5D).

### Effects of AsA on the antioxidant system of the NaCl-treated seedlings

**Fig 6:**
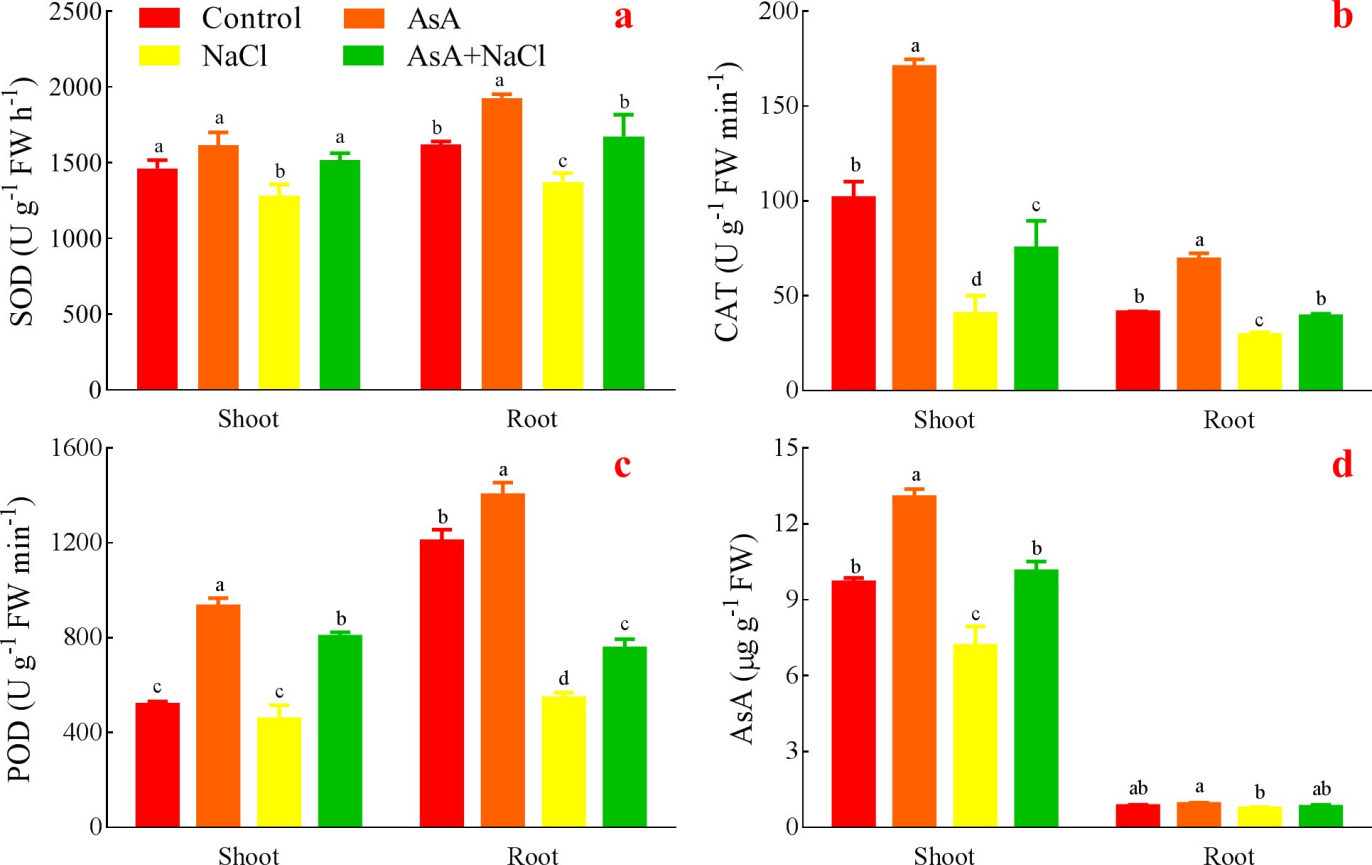
Effects of AsA on SOD (a), CAT (b), POD (c), and endogenous AsA (d).

The AsA treatment showed the highest SOD activity both in the shoots and roots. SOD activity of the NaCl treatment was inhibited, which was significantly lower than that of the AsA+NaCl treatment. Even the AsA+NaCl treatment had higher SOD activity than the control (Fig 6A).

Both in the shoots and roots, CAT activity of the AsA treatment had a significant maximum, while the NaCl treatment had a significant minimum. The AsA+NaCl treatment increased CAT activity compared with the NaCl treatment, and it occurred significant differences (Fig 6B).

POD activity in the AsA treatment was dramatically higher than the others both in the shoots and roots (Fig 6C). The NaCl treatment suppressed POD activity and had the minimum. Moreover, the AsA+NaCl treatment significantly promoted the activity of POD compared with the NaCl treatment.

The AsA treatment had the best effect on endogenous AsA both in the shoots and roots (Fig 6D). The NaCl treatment inhibited endogenous AsA, and had the minimum. The AsA+NaCl treatment increased endogenous AsA contents of shoots and roots.

### Effects of AsA on Na^+^, K^+^, Cl^-^ of the NaCl-treated seedlings

**Fig 7:**
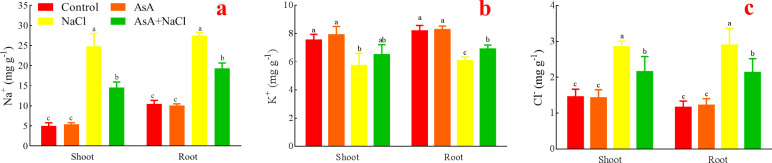
Effects of AsA on Na+ (a), K+ (b), and Cl^¯^ (c).

The NaCl treatment significantly increased Na^+^ level both in the shoots and roots. There was no difference between the AsA treatment and the control. However, the AsA+NaCl treatment dramatically decreased Na^+^ level (Fig 7A).

The AsA treatment had no significant increase in K^+^ compared with the control. The NaCl treatment had a minimum of K^+^. Whereas the AsA+NaCl treatment increased K^+^ level both in the shoots and roots (Fig 7B).

There was no significant difference occurred in Cl^-^ level between the control and the AsA treatment. The NaCl treatment remarkably increased Cl^-^, while the AsA+NaCl treatment decreased it (Fig 7C).

### Principal component analysis (PCA) of main parameters

**Fig 8:**
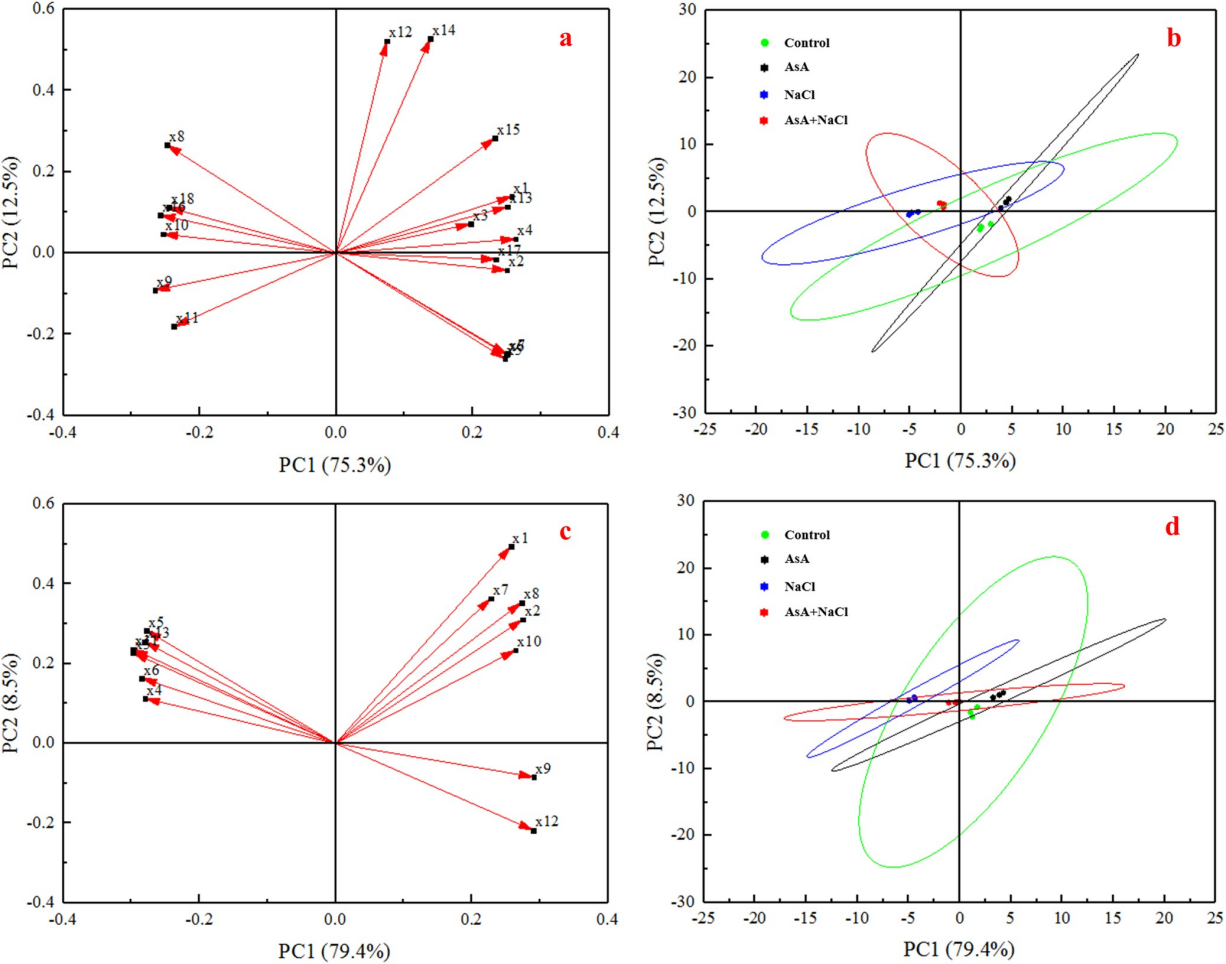
The loading plots (a and c) and score plots (b and d) of shoot and root, respectively. In Fig 8A, x1-x18 were shoot length, FW, DW, Chl a, Chl b, Chl a+b, Cx+c, RC, MDA, H_2_O_2_, O_2_·^¯^, SOD, CAT, POD, AsA, Na^+^, K^+^, and Cl^¯^ of shoots, respectively. In Fig 8C, x1-x13 were FW, DW, RC, MDA, H_2_O_2_, O_2_·^¯^, SOD, CAT, POD, AsA, Na^+^, K^+^, and Cl^¯^ of roots, respectively.

The PCA indicates that the 18 parameters in the shoots were divided into PC 1 (75.3%) and PC 2 (12.5%). The RC (x8), MDA (x9), H_2_O_2_ (x10), O_2_·^¯^ (x11), Na^+^ (x16), and Cl^¯^ (x18) were distributed in the second and third quadrants, and almost all of them showed the opposite relationships to the others distributed in the first and fourth quadrants (Fig 8A). All four groups had intersecting sections, which meant these 18 parameters completely could be used as an explanation for salt-tolerance metabolism (Fig 8B).

The loading plot reveals that these 13 parameters in the roots were divided into PC 1 (79.4%) and PC 2 (8.5%), which showed a similar relationship with the shoots (Fig 8C). However, the score plot indicates that there was no shared portion between the AsA treatment and the NaCl treatment (Fig 8D).

## Discussion

Numerous studies have demonstrated that AsA is involved in senescence regulation [31], photosynthetic structure protection [32], glycometabolism [33], floral induction [34], ion transportation [35], and stress responses [36]. However, it is difficult to ascertain that what dose of AsA is better because the effectiveness varied with a stage of plant growth, mode of AsA application, genetic architecture of plant species, and type of stress [36]. As a natural substrate of peroxidases, AsA chemically reacts with ROS and works in gene modulation, enzyme regulation, and plant growth [36]. So far, five AsA biosynthetic pathways in plants have been elucidated, and the L-galactose pathway is the main biosynthetic pathway for AsA [37–39]. In the L-galactose pathway, L-galactose-1, 4-lactone dehydrogenase (GLDH) is the key enzyme in the last step of the AsA biosynthesis pathway, and it oxidizes L-galactose to AsA [39–42]. Priming refers that the hydration state of seeds is controlled so that seeds can avoid the endoderm to break through the seed coat and be able to produce pre-germination metabolism. Thus, it improves the vigor of seeds and the growth potential of seedlings under different ecological conditions [43]. Seed germination is a key developmental process [44], and the priming treated with appropriate small molecular biological active substances can not only initiate the metabolic reaction before germination but also participate in the metabolic reaction during germination. Moreover, in the initial stage of germination, the nutrients and energy sources are mainly from the oxidative decomposition of starch and protein by amylase and protease. In the present study, the optimal AsA promoted germination, growth, and AsA (Figs 1–3). Exogenous AsA likely enhances GLDH activity, thus improves seed AsA, which is consistent with the positive effects of AsA application in wheat [45]. Likewise, the addition of AsA improved seed germination and increased acid phosphates activity, chlorophyll content, and dry mass of alfalfa [46]. Furthermore, appropriate AsA can improve amylase and protease activities to hydrolyze starch and protein for germination. However, the high concentrations of AsA inhibited the seed germination and suppressed the seedling growth, even 100 mmol L^-1^ AsA caused serious rot (Figs 1–3). Similarly, the increased level of exogenous ascorbate can inhibit germination through ROS elimination which results in the failure of germination initiation [47]. ROS, such as H_2_O_2_, O_2_·^¯^ and hydroxyl radical (OH·), can promote oxidative and peroxidative reactions. The study has indicated that *Hydrocharis dubia* seeds maintained high seed vigor in ambient wet storage condition through scavenging H_2_O_2_ by the antioxidant systems [48]. So, the proper level of ROS is a signal to induce and improve seed germination, while too low levels are not enough to induce germination and too high levels affect germination by oxidative damage. Thus, maybe excessive AsA thoroughly scavenges ROS and results in a too low level of ROS, finally resulting in a low germination. Even studies have revealed that the resistance of the deletion mutant of AsA to pathogenic bacteria was significantly increased [49,50]. Moreover, it has been found that the enhanced resistance is associated with H_2_O_2_, SA, and *NPR1* gene [50]. Excessive AsA inhibited amylase and protease activities and caused starch and protein to rot during incubation (Fig 2). Hence, it indicates that the high concentration of AsA can cause low germination and serious rot of alfalfa seeds.

Apoplastic ascorbate contents are important for environmental stress perception and thus are involved in the subsequent downstream stress signaling and responses in plants [51]. It reported that redox states of the apoplastic ascorbate levels influence hormonal balance, growth responses, mitogen-activated protein kinase (MAPK) signaling cascades, and antioxidant system; while glutathione levels almost remain unaffected [52]. Due to its apoplastic localization, AsA constitutes an important role in stress perception, redox homeostasis, and subsequent regulation of oxidative stress and physio-biochemical responses both under normal conditions and abiotic stresses [36]. Its role in plants under stress conditions has become crucial because of its multipurpose involvement in plant cells. In addition, AsA can improve tolerance against abiotic stresses by enhancing plant growth, photosynthesis rate, transpiration, oxidative defense potential, and photosynthetic pigments [36,53]. Hence, AsA application increased length, FW and DW, even Chl a, Chl b, Chl a+b, and Cx+c contents of the seedlings under NaCl stress (Fig 4).

The levels of AsA in most plant species are not adequate to effectively mitigate the adverse impacts of stress. In such cases, AsA application is desirable. Exogenous AsA is not only helpful in improving the yield and growth of plants by regulating different physio-biochemical pathways under non-stress conditions, but also stress conditions such as salinity, drought, and temperature extremes [36]. Studies revealed that AsA application improves growth, endogenous AsA, and activities of SOD, CAT, and POD, while it decreases ion leakage and lipid peroxidation under stresses [36,45]. What’s more, the most efficient role of exogenous AsA is to protect lipids and proteins against salinity or drought-induced oxidative adversaries [36]. Similarly, the levels of RC, MDA, H_2_O_2,_ and O_2_·^¯^ increased while the activities of the antioxidant system decreased in the NaCl treatment, which was opposite in the AsA treatment and the AsA+NaCl treatment (Figs 5 and 6). AsA is known to protect organelles and cells from ROS, which over-accumulates because of the stress-induced oxidative damages in plants [54,55]. Oxidative stress is a consequence of an imbalance between the formation of free oxygen radicals and their inactivation by the antioxidant defense system [56]. The oxidants are capable of causing damage to various cellular and extracellular constituents. These effects generally appear after exposure to a relatively high concentration of ROS and/or a decrease in the antioxidant defense system against ROS [18,57]. That is, oxidative stress always produces high ROS, whereas the antioxidant system reduces ROS to alleviate stress-induced oxidative damages. Besides, the balance between generation and elimination of ROS is extremely crucial for the survival and growth of plants under adverse environments [18]. However, serious oxidative damages can also reduce or inhibit the effects of the antioxidant system. As one of the various non-enzymatic antioxidants, AsA occurs ubiquitously in plants and plays a vital role in growth and metabolism. Exogenous AsA is necessary and advisable when endogenous AsA is not adequate to alleviate the stresses [54,55,58]. Exogenous AsA has a positive effect on alleviating the adverse effects of salinity stress and improving salt tolerance by regulating a myriad of biological processes. Similarly, foliar-applied AsA plays a part in up-regulating the activities of the oxidative defense system in *Lymonium stocksii* under saline stress [59]. High accumulation of AsA is related to stress tolerance because AsA can scavenge ROS and upgrade the oxidative defense potential, thus improving the growth and development of plants under stress conditions [36]. And the excessive ROS produced under high salinity was scavenged by the increased levels of antioxidant enzymes and corresponding antioxidants in heteromorphic seeds [60]. AsA pretreatment could partially alleviate the harmful effects of salinity by increasing vigor, antioxidative enzyme activities, and accumulation of osmolytes in wheat [61].

The adverse effects of salt stress on plants include osmotic stress, ionic stress, and secondary stress [62,63]. If too much salt gets into the transpiration stream of a plant, the cells in the leaves and even plant growth will be affected, which is called the ion surplus effect. Secondly, when the salt level reaches the threshold, ion toxicity will occur. Beyond this threshold, plants cannot maintain ion balance and cause secondary reactions such as oxidative stress [64]. That is, both osmotic stress and ion accumulation stimulate ROS, which induces cytoplasmic membrane damage and metabolic dysfunction of plants [65,66]. Excessive Na^+^ or Cl^¯^ can inhibit photosynthesis and directly affect plant health (Figs 4 and 7). What’s more, K^+^ can also be reduced (Fig 7B). Because the high concentration of Na^+^ mainly reduces uptake of K^+^ and Ca^2+^ by reducing the stomatal conductance of plants, thus inhibiting photosynthesis. While a high concentration of Cl^¯^ inhibits photosynthetic capacity due to the non-stomatal effect and chlorophyll degradation. The lack of K^+^ in plants has a great impact on growth and development. Therefore, maintaining a stable K^+^ concentration in plant cytoplasm under high Na^+^ concentration is a key factor for plants to resist salt stress. Furthermore, leaf mesophyll K^+^ and Cl^¯^ fluxes and ROS production can be used to predict rice salt tolerance at the reproductive stage in the greenhouse and field conditions [67]. Under NaCl stress, AsA could reduce Na^+^ and Cl^¯^, and increase K^+^, simultaneously enhance the antioxidant system, thereby improving the salt-tolerance of alfalfa. Although AsA could enhance the growth of the seedlings without NaCl stress, it had no remarkable difference in ions. It indicates that AsA mainly plays a role in modulating ions and mitigating ion damage in alfalfa under stress conditions.

As to the parameters of shoots and roots, PCA consisted of two main components and can well interpret the results (Fig 8). Combined with the results of PCA, it can be observed that the AsA treatment can improve the growth of seedlings, reduce the adverse impacts of ion penetration, and increase antioxidant levels, thus alleviating lipid peroxidation and excessive ROS induced by NaCl stress. Whereupon, the seedlings can grow better under salt stress.

## Conclusions

A low concentration of exogenous AsA can promote seed germination and improve seedling growth of alfalfa, and the optimum concentration is 0.1 mmol L^-1^ AsA; whereas the higher concentrations of AsA not only inhibit seed germination and growth but also cause serious rot. Exogenous AsA can enrich endogenous AsA of seedlings, while its positive effects reduce with the increase of exogenous concentration. AsA inhibits membrane permeability, MDA, H_2_O_2,_ and O_2_·^¯^ by increasing antioxidant system, and decreases ion toxicity, thereby enhancing the salt-tolerance and promoting the growth of alfalfa. The parameters were divided into two main components both in the shoots and roots. It is suggested that the optimum AsA has the potential to treat alfalfa for promoting seed germination and seedling salt-tolerance.

## Supporting information

S1 File (XLSX)
